# Characteristic Volatile Organic Compound Analysis of Different Cistanches Based on HS-GC-IMS

**DOI:** 10.3390/molecules27206789

**Published:** 2022-10-11

**Authors:** Shiqi Zhou, Duo Feng, Yaxi Zhou, Hao Duan, Yue He, Yongjun Jiang, Wenjie Yan

**Affiliations:** 1 College of Biochemical Engineering, Beijing Union University, No. 18, Chaoyang District 3, Futou, Beijing 100023, China; 2 Beijing Key Laboratory of Bioactive Substances and Functional Food, College of Biochemical Engineering, Beijing Union University, No. 18, Chaoyang District 3, Futou, Beijing 100023, China; 3 Inner Mongolia Sankou Biotechnology Co., Ltd., Ordos 017000, China

**Keywords:** oil cistanche, blood cistanche, cistanche tubulosa in Xinjiang, HS-GC-IMS, VOCs

## Abstract

Cistanche is a medicinal and food homologous substance with a long history of consumption and medicinal use in China. In order to further understand the volatile organic compound differences between different cistanches, this study selected oil cistanche, blood cistanche and cistanche tubulosa in Xinjiang for HS-GC-IMS volatile organic compounds, and established the characteristic fingerprints of different cistanches for organic content and characteristic organic compound analysis. PCA and cluster analysis were used to study the similarity between different cistanches. After qualitative analysis, a total of 32 volatile organic compounds were identified, covering aldehydes (17), ketones (5), furans (1), alcohols (5), lactones (1) and esters (3), and the volatile organic compounds between samples a, b and c could be significantly distinguished, affecting the flavor of cistanche itself. It provides a basic theoretical basis for the study of cistanche flavor.

## 1. Introduction

Cistanche grows in arid environments such as deserts and is a parasitic plant, often parasitic at the root of sophora or red willow, known as “desert ginseng” [1]. In 2016, the Expert Review Committee of the China National Center for Food Safety Risk Assessment (CFSA) reviewed it in accordance with statutory procedures and finally found that Alxa Desert Cistanche met the food safety requirements. In 2018, the desert cistanche (*Cistanche deserticola Ma*) was included in China’s Catalogue of Substances that are Both Food and Chinese Medicinal Materials according to Tradition [2]. In 2020, the National Health Commission and the State Administration of Market Supervision jointly issued the “Notice”, proposing to carry out pilot work on the production and operation of nine substances such as cistanche in accordance with the tradition of both food and Chinese medicinal materials (hereinafter referred to as food and medicine substances), and since then, cistanche has officially begun to be used in ordinary food production as a new raw food material [3]. However, the sharp increase in market demand, the lack of resources and the uneven quality of cistanche in the existing market has brought great inconvenience to the supervision of regulatory authorities.

*Cistanche deserticola Y.C.Ma* and *Cistanche tubulosa (Schenk) Wight* is the most-studied cistanche category, currently using inductively coupled plasma atomic emission spectrometry (ICP-AES), high performance liquid chromatography (HPLC), LTQ-Orbitrap-based (linear ion trap-Orbitrap-based) strategy, infrared fingerprint analysis soft independent modeling of class analogy (SIMCA) and genomics [4,5,6,7,8] and other methods to carry out variety identification and quality identification. Among them, the section of tubulosa cistanche is gray-brown, irregular in shape, with brown dot distribution, and the content of phenethyl alcohol glycoside is higher than that of other varieties, which has research potential in preventing hyperglycemia and in treating hyperlipidemia [9,10]. Cistanche can be harvested two seasons a year, and because the appearance of cistanche harvested in autumn is oilier and thicker, it is called oil cistanche. Some cistanche cross-sections differ from the tan of ordinary cistanche, which is known as blood cistanche by the local people in Inner Mongolia. Studies show that oil cistanche is a stable variant of the desert cistanche; the fresh oil cistanche stem is lilac and soft in texture, and the appearance looks oily. After slicing, the cistanche is more transparent, and after drying, the surface is reddish brown, the section is black brown, and the sliced surface is neatly arranged as radial or wavy, with a slight sweetness [11,12]. At the same time, it was found that oil cistanche was found to have a higher content of echinacein and mullein glycosides than non-oil cistanche with the same growth cycle [13]. Echinacein and mullein glycosides are phenylethanol glycosides, playing an active role in antioxidation, hypoglycemia and blood lipids, and have anti-inflammatory properties [10]. These properties may enhance the anti-aging and anti-fatigue effects of oil cistanche. However, blood cistanche is not a separate variety, but its cross-section is purple, hence it being known as blood cistanche. After research and analysis, it was found that the ether terpene content of blood cistanche may differ from other cistanches.

Gas chromatography–ion mobility spectroscopy (GC-IMS) has the advantages of efficient separation and rapid response, which can quickly analyze the types of volatile organic compounds (VOCs) in the sample, which helps to identify counterfeit and inferior cistanche and the analysis of characteristic flavor compounds. In recent years, headspace-gas chromatography–ion mobility spectrometry (HS-GC-IMS) has been widely used in the quality identification and flavor difference analysis of food and drugs, which has played an important role in food safety supervision and the quality monitoring of the production process [14]. Some examples of its use include the difference analysis of the flavor of smoked chicken in different origins [15], the quality control of fresh noodle storage flavor [16], the relationship between the flavor and concentration of distilled wine [17], the analysis of characteristic flavor substances of the fermentation process of northeast sauerkraut [18], and the effects of varieties and cooking on the characteristic volatile compounds of sorghum [19]. These studies show that HS-GC-IMS plays an important role in the analysis of the characteristic VOC of samples.

Thus, in our research, we used the HS-GS-IMS method to analyze and study the characteristic VOCs in oil cistanche, blood cistanche and tubulosa cistanche (in Xinjiang). On this basis, we combined principal component analysis (PCA) and similarity evaluation to analyze the characteristic VOCs in cistanche. Understanding the differences among oil cistanche, blood cistanche and tubulosa cistanche (in Xinjiang) will provide a theoretical basis for the flavor analysis and quality identification.

## 2. Results

### 2.1. HS-GC-IMS Topographic Plots of Different Cistanches

**Figure 1:**
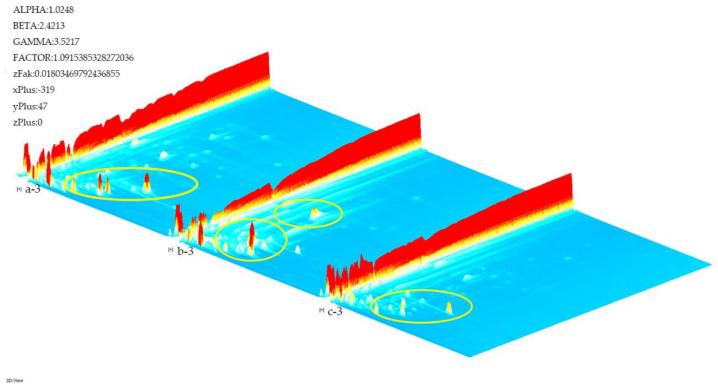
3D topographic plots. a: Oil cistanche, b: blood cistanche, c: cistanche tubulosa in Xinjiang.

HS-GC-IMS was adopted to identify the volatile organic compounds in different cistanche samples. As seen in Figure 1, 3D topographical visualization was displayed using the Reporter plug-ins to ascertain the differences between cistanches. From Figure 1, it can be seen that there are significant differences in the VOCs between a, b and c by combining the retention time, migration time and peak intensity. This is shown in a yellow circle (Figure 1). When comparing oil cistanche and blood cistanche with cistanche tubulosa in Xinjiang, their VOCs are more abundant; it can be inferred from this that VOCs may affect the purple facets of oil cistanche and blood cistanche, giving them different morphologies.

**Figure 2:**
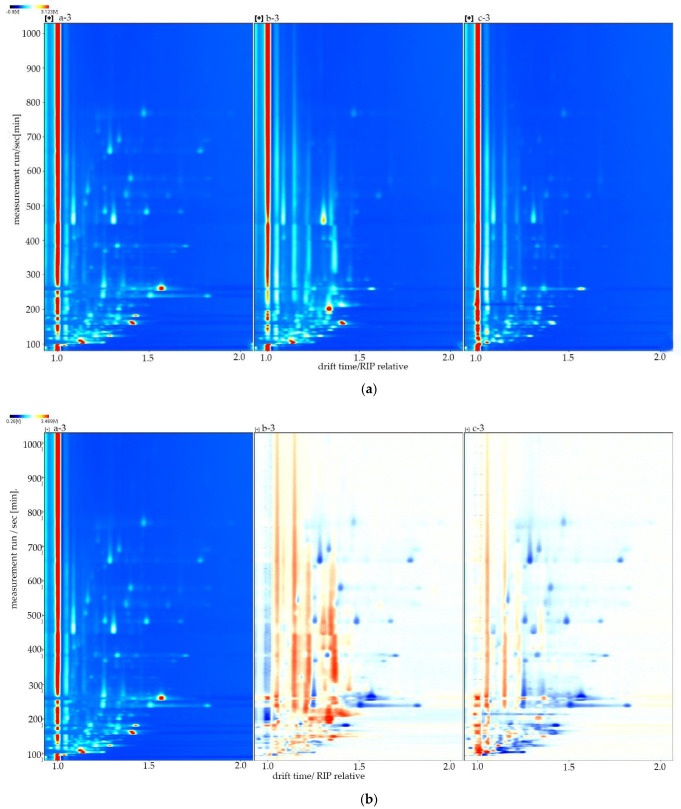
(**a**) Topographic plot of all samples. (**b**) GC-IMS of the sample Difference plot. a: Oil cistanche, b: blood cistanche, c: cistanche tubulosa in Xinjiang.

The three-dimensional spectrum can only look at the differences between the cistanche samples at a cursory level. So we further refined the flavor differences between the samples through a top-down comparative analysis [20]. The top view, as a background, is mainly blue, the abscissa is the ion migration time (normalized), and the ordinate coordinate is the retention time (s) of the gas chromatography. The red vertical line is the reactive ion peak (RIP, normalized), and this line is the horizontal coordinate 1.0. Each point of the right area of the RIP represents a VOC. The color represents the intensity of the signal peak: white is the lower intensity, and as the color deepens, this means that the signal is stronger. The darker the red, the stronger the signal. As illustrated in Figure 2a, most signals occur at a retention time of 100 to 800 s and a drift time of 1.0 to 1.6 ms. Cistanche had strong signal peaks at a drift time of 1.2 and 1.4~1.6 ms, and blood cistanche had strong signal peaks at a drift time of 1.1~1.2 and 1.3~1.4 ms, indicating that the distribution of compounds in the region may be characteristic of VOCs. In the next step, we selected sample a as the reference, deducted the reference treatment on the other two sample spectra, and finally obtained a sample difference comparison map (Figure 2b). The white background in Figure 2b indicates that the VOCs are the same, red indicates that the content of the substance is higher than the reference, and blue indicates that the content of the substance is lower than the reference. The darker the color, the greater the concentration difference. Compared with sample a, sample b had a drift time of 1.0~1.5 ms, and the concentration of most substances was significantly higher than that of sample a. Sample c had a drift time of 1.0~1.2 ms compared with sample a. Through the analysis of the results, it can be seen that most of the VOC content in the oil cistanche and blood cistanche are higher than those of tubulosa cistanche (in Xinjiang), which may be one of the main reasons for the sweet taste of oil cistanche.

### 2.2. Identification of Volatile Compounds from Different Cistanches

**Figure 3:**
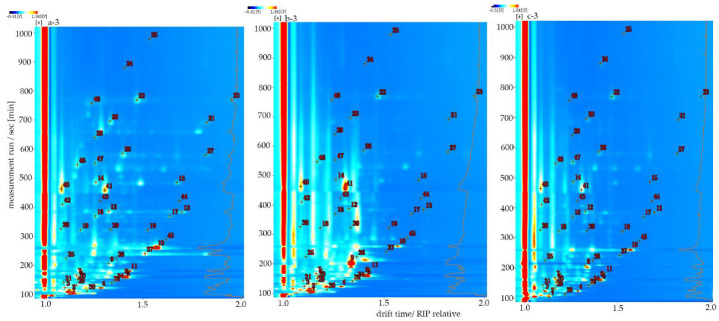
HS-GC-IMS spectra of different cistanches. The numbers are identified volatile compounds. a: Oil cistanche, b: blood cistanche, c: cistanche tubulosa in Xinjiang.

To further understand the differences between VOCs in different cistanches, the spectra of the sample were qualitatively analyzed using VOCal software. As shown in Figure 3, the abscissa and ordinate coordinates were respectively used to denote the drift time (normalized) and reaction time (s). Each red number corresponds to an identified VOC in Table 1. It can be clearly seen from Figure 3 that a total of 48 peaks were identified in this study, of which 16 VOCs were of both monomeric and dimer forms, so a total of 32 VOCs were identified from cistanche, mainly including ketones, alcohols, aldehydes, furans, lactones and esters. After comparative analysis, it was found that some compounds may have multiple signals or spots [21]. This suggests that these chemicals are in higher concentrations and dimers are formed in IMS drift tubes, which are methylpropanal, 3-methylbutanal, 2-methylbutanal, heptanal, (E)-2-heptenal, 2-heptanone, (E)-2-hexenal, (E)-2-octenal, n-nonanal, octanal, ethyl acetate, 2-methylpropanol, (E)-2-pentenal, furfural, gamma-butyrolactone and methyl hexanoate. Combined with Figure 2, we can see that ethanol (strong alcohol), acetone (ethereal solvent apple pear) and 2-methylpropanol (ethereal winey cortex) are relatively high in samples a and b, and they will help to add a special flavor to the oil cistanche and blood cistanche, making them emit a fruity aroma.

### 2.3. Gallery Plots of Different Cistanches

**Figure 4:**
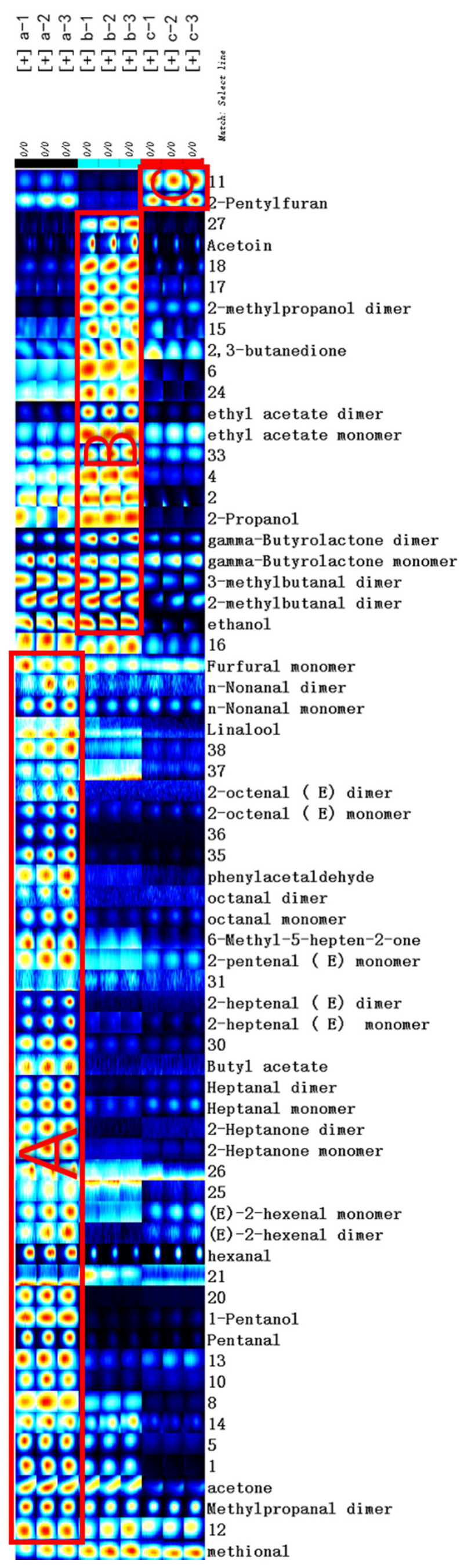
Fingerprint of VOCs of different cistanches. a: Oil cistanche, b: blood cistanche, c: cistanche tubulosa in Xinjiang.

For the further analysis of characteristic VOCs in samples a, b and c, all peaks of each sample were selected for fingerprint comparison (Figure 4). The fingerprint comparison analysis between samples was carried out using the Gallery Plot plug-in. Each row represented all signal peaks detected in the same sample, each column represented the signal intensity of the same VOC in different samples, and the light and shade of the point color reflected the amount of the substance in the sample. The brighter the color, the higher the VOC content. The number on the ordinate indicates that the sample has been detected but has not yet been characterized.

As shown in Figure 4, there were obvious differences in the VOCs between the samples, with the most volatile substances in sample a and most of the concentrations being higher. Sample c had the fewest types of volatile substances and relatively low content. The main components of the A region corresponding to sample a are methylpropanal, acetone, pentanal, valeraldehyde, hexanal, (E)-2-hexenal, 2-heptanone, heptanal, butyl acetate, (E)-2-heptenal, (E)-2-pentenal, 6-methyl-5-hepten-2-one, octanal, phenylacetaldehyde, (E)-2-octenal, linalool, nonanal and furfural. The B region corresponding to sample b is mainly ethanol, 2-methypropanol, 3-methylbutanal, gamma-butyrolactone, propanol, ethyl acetate, 2,3-butanedione, 2-methylbutanal, acetoin and other substances that are dominant. The characteristic VOC of sample c is 2-pentylfuran.

In sample a, the main characteristic VOCs are ketones and aldehydes. Among them, aldehydes account for a relatively high proportion of fruits and vegetables, which will bring unique aromas and flavors [22]. Hexanal, for example, smells like citrus and orange with a fresh, lingering aftertaste; pentanal smells like cocoa and chocolate notes; furfural smells sweet, woody, bready and caramellic. Ketones and esters also bring a unique flavor, increasing the sweetness and freshness of the substance [23]. For example, butyl acetate has a sweet ripe banana, tutti frutti and candy-like odor, while linalool has a citrus, orange, lemon, floral, waxy, aldehydic and woody odor. It can be inferred that oil cistanche is more sweet than general cistanche, mainly affected by ketones and aldehydes. There are more alcohols and esters in sample b, including gamma-butyrolactone (milky and creamy with fruity peach-like afternotes), propanol (with a slightly sweet and fruity nuance of apple and pear) and ethyl acetate (ethereal, fruity, sweet, with a grape and cherry nuance). They have a fresh, fruity aroma, so the flavor of blood cistanche may be fresher than that of ordinary cistanche. The main taste of cistanche tubulosa (in Xinjiang) is woody, which is likely related to 2-pentylfuran (green, waxy, musty, cooked caramellic flavor).

### 2.4. Clustering Analysis of Different Cistanches

#### 2.4.1. Dynamic PCA of Samples

Principal component analysis, a statistical analysis technology of multiple variates, is always used in fingerprint research. As an unsupervised pattern recognition method, it preserves the variability of the original data, mainly by reducing the dimension of the dataset and retaining the variability of the original data through a linear combination of variables [24]. In this study, the signal peak area corresponding to each sample’s VOCs was normalized using the Dynamic PCA plug-in. Then, the principal components are determined and the corresponding contribution rate calculations are performed [25]. Finally, the PCA sample analysis plot is drawn to determine the regularity and difference of volatile compounds between samples.

**Figure 5:**
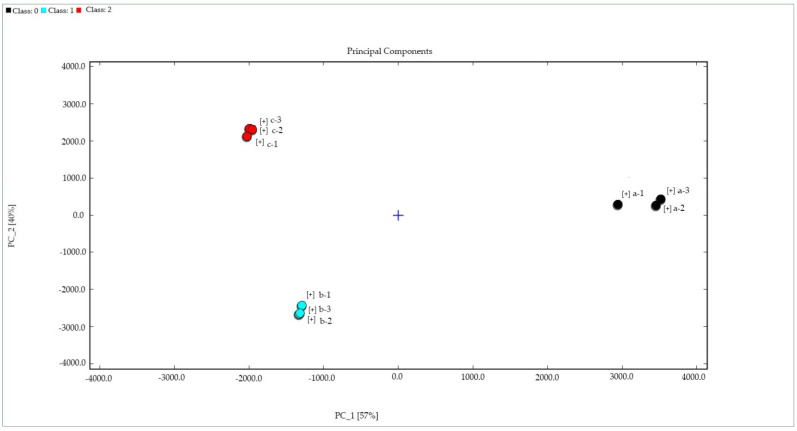
PCA of different samples. a: Oil cistanche, b: blood cistanche, c: cistanche tubulosa in Xinjiang.

Through the principal component analysis of the sample, it can be found that the PC1 contribution rate is 57%, the PC2 contribution rate is 40%, and the cumulative total contribution rate is 97%. It is generally believed that when the cumulative contribution rate of PC1 and PC2 is higher than 60%, the principal component analysis model is a high-quality model [26]. Therefore, the PCA model is a high-quality model that can be used to analyze the differences in VOCs between different cistanches. As shown in Figure 5, sample a is located in the positive fraction value of PC1 and the central axis area of PC2, sample b is located in the negative score area of PC1 and PC2, and sample c is located in the negative score area of PC1 and the positive score area of PC2; the distinction between the three is obvious. Wu et al. applied PCA to successfully elucidate the characteristics of four different sources of honey in China as one of the bases for judging the distinction between honey [27]. Li et al. used PCA and found that the flavor differences between fresh and dried Tricholoma matsutake samples were large, and the regional distribution also affected the flavor of Tricholoma matsutake [21]. Therefore, based on the VOC content in Figure 4, PCA can be applied and distinguished from the quality identification of different kinds of cistanche and cistanche of the same species. However, PCA needs to standardize the data, and the transformed matrix must be a square matrix. Different people may have different processing results, which reduces the reliability of the data. Therefore, we also need to improve the analysis method in follow-up research.

#### 2.4.2. Fingerprint Similarity Analysis Using Euclidean Distance

**Figure 6:**
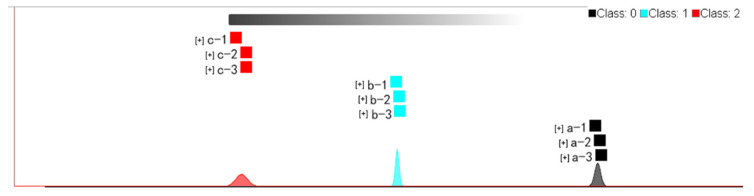
Fingerprint similarity based on Euclidean distance of different samples. a: Oil cistanche, b: blood cistanche, c: cistanche tubulosa in Xinjiang.

From Figure 6 and Table 2, the similarity of the fingerprints of VOCs in cistanche can be concluded according to the distance. From Figure 6, it is clear that the distance between samples a and c is the farthest, followed by samples a and b, and the distance between samples b and c is relatively close. It can be seen from the numerical calculation of Table 2 that the average Euclidean distance between samples a and b is 11,937,541.855, the average Euclidean distance of samples a and c is 12,278,785.505, and the average Euclidean distance of samples b and c is 8,816,334.693. Therefore, it can be considered that the difference between oil cistanche and cistanche tubulosa (in Xinjiang) is more significant, and the similarity of the fingerprints is not high, while blood cistanche and cistanche tubulosa (in Xinjiang) are close to each other and have fingerprint similarity.

## 3. Materials and Methods

### 3.1. Materials

The fresh oil cistanche, blood cistanche and cistanche tubulosa (in Xinjiang) in the experiment were provided by Inner Mongolia Sankou Biotechnology Co., Ltd., Ordos, China.

First, the fresh cistanche was washed to remove impurities, cut into small pieces and put into a beater for wet crushing. The initial crushed powder was tipped out and dried until the water content was less than 5%, and then secondary crushing was carried out. Finally, the collected powder was packaged and sterilized and stored in a dry environment for later use.

### 3.2. HS-GC-IMS

Gas-phase ion mobility spectrum: FlavourSpec^®^ (the G.A.S. Department of Shandong Hai Neng Science Instrument Co., Ltd., Dezhou, Shan-dong, China).

Column type: MXT-5 (L-15 m, ID-0.53 mm, FT-4 μm) with column temperature maintained at 60 °C.

Carrier gas/drift gas: N_2_ (purity ≥ 99.999%).

Gas chromatography conditions: 0–2 min-2 mL/min; 2–10 min-2–10 mL/min; 10–20 min-10–100 mL, and after 20 min, the analysis was stopped. IMS temperature set to 45 °C, N_2_ flow rate of 150 mL/min.

Sample handling: Take 0.5 g of powder in a 20 mL headspace flask and incubate at 70 °C for 20 min at 500 rpm. The temperature of the injection needle was 85 °C, with the injection volume set to 200 μL [28].

### 3.3. Data Analysis

The analysis software that accompanies the HS-GC-IMS instrument includes VOCal and three plug-ins (Reporter, Gallery Plot and Dynamic PCA plug-ins). VOCal was used to view analytical spectra and perform the qualitative quantification of data, which is mainly retrieved through the NIST database and IMS database built into the application software. The Reporter plug-in is used to directly compare spectral differences between samples. Fingerprint comparison using the Galaxy Plot plug-in visually and quantitatively compares VOC differences between different samples. The Dynamic PCA plugin was used to cluster the samples and quickly determine the type of unknown samples. The Euclidean distances were calculated by selecting the strength of the compounds in the evaluation area to quickly compare the samples, using the software’s own algorithms.

The PCA data matrix for the statistical analysis of the results of the chromatographic tests had 38 columns (names of volatile compounds) and nine rows (samples of different types of cistanche). The input matrix was scaled automatically.

## 4. Conclusions

In this study, a total of 48 signal peaks from topographic plots were detected in different cistanches using HS-GC-IMS. A total of 32 signal peaks were identified that belong to six categories: ketones, alcohols, aldehydes, furans, lactones and esters. From the fingerprint results, it can be observed that different types of cistanche affect the concentration of these compounds, giving them different flavors. From the characteristic volatile organic compound results, it is clear that the esters and ketones in the oil cistanche increase the sweeter woody and fresh flavor, the alcoholic and ester substances in the blood cistanche add to the fruity flavor. The tubulosa cistanche (in Xinjiang) is more woody and green, which may be one of the reasons why the oil cistanche smells sweeter. In addition, by using visualization methods, such as principal component analysis and Euclidean distance clustering analysis, the flavor differences between the different cistanches can be clearly highlighted, which can be used as one of the bases for detecting and analyzing cistanche varieties and can provide basic testing guidance for standardized market management. These analyses show that HS-GC-IMS, PCA and clustering analysis are ideal methods for studying the VOC composition of cistanche and identifying different cistanches. Moreover, the results of this study help to understand the aromatic characteristics of oil cistanche, blood cistanche and cistanche tubulosa in Xinjiang, which is helpful for cistanche quality analysis and product development applications. However, due to the limited types of compounds contained in the NIST database and IMS database built into the qualitative analysis software, 38 signal peaks have not yet been characterized. So, the database can be expanded in combination with HPLC, GC and MS methods to further understand the aromatic characteristics of cistanche, providing a reliable theoretical basis for cistanche in quality testing and the development and application of ordinary foods.

## Figures and Tables

**Table 1:** Qualitative results of different cistanches.

Count	Compound	CAS#	Formula	MW	RI	Rt (s)	Dt (RIPrel)	Comment
1	Ethanol	C64-17-5	C_2_H_6_O	46.1	519.7	102.932	1.13339	
2	Acetone	C67-64-1	C_3_H_6_O	58.1	537.7	110.795	1.13243	
3	Methylpropanal	C78-84-2	C_4_H_8_O	72.1	567.8	123.954	1.10451	monomer
4	Methylpropanal	C78-84-2	C_4_H_8_O	72.1	567.1	123.633	1.28451	dimer
5	3-Methylbutanal	C590-86-3	C_5_H_10_O	86.1	647.2	158.616	1.17285	monomer
6	3-Methylbutanalr	C590-86-3	C_5_H_10_O	86.1	645.7	157.974	1.40964	dimer
7	2-Methylbutanal	C96-17-3	C_5_H_10_O	86.1	667	167.281	1.16323	monomer
8	2-Methylbutanalr	C96-17-3	C_5_H_10_O	86.1	662.2	165.195	1.40001	dimer
9	Acetoin	C513-86-0	C_4_H_8_O_2_	88.1	726.1	206.658	1.32704	
10	Hexanal	C66-25-1	C_6_H_12_O	100.2	793.2	262.856	1.56653	
11	Pentanal	C110-62-3	C_5_H_10_O	86.1	696.6	183.055	1.4304	
12	Heptanal	C111-71-7	C_7_H_14_O	114.2	900.4	386.492	1.32578	monomer
13	Heptanal	C111-71-7	C_7_H_14_O	114.2	898.8	383.682	1.69888	dimer
14	(E)-2-Heptenal	C18829-55-5	C_7_H_12_O	112.2	956.7	486.525	1.2615	monomer
15	(E)-2-Heptenal	C18829-55-5	C_7_H_12_O	112.2	955.8	484.839	1.67241	dimer
16	2-Heptanone	C110-43-0	C_7_H_14_O	114.2	890.6	370.195	1.25898	monomer
17	2-Heptanone	C110-43-0	C_7_H_14_O	114.2	890.1	369.633	1.63838	dimer
18	(E)-2-Hexenal	C6728-26-3	C_6_H_10_O	98.1	846.7	321.864	1.18335	monomer
19	(E)-2-Hexenal	C6728-26-3	C_6_H_10_O	98.1	846.7	321.864	1.52494	dimer
20	(E)-2-Octenal	C2548-87-0	C_8_H_14_O	126.2	1067.7	695.821	1.33298	monomer
21	(E)-2-Octenal	C2548-87-0	C_8_H_14_O	126.2	1065.5	691.686	1.82433	dimer
22	n-Nonanal	C124-19-6	C_9_H_18_O	142.2	1105.4	769.423	1.46964	monomer
23	n-Nonanal	C124-19-6	C_9_H_18_O	142.2	1105.9	770.25	1.95024	dimer
24	(E)-2-Nonenal	C18829-56-6	C_9_H_16_O	140.2	1161.8	879.239	1.40734	
25	Decanal	C112-31-2	C_10_H_20_O	156.3	1212.8	978.677	1.53391	
26	Octanal	C124-13-0	C_8_H_16_O	128.2	1010.8	585.11	1.39729	monomer
27	Octanal	C124-13-0	C_8_H_16_O	128.2	1008.1	579.876	1.81921	dimer
28	Phenylacetaldehyde	C122-78-1	C_8_H_8_O	120.2	1038.8	639.539	1.25666	
29	2,3-Butanedione	C431-03-8	C_4_H_6_O_2_	86.1	586.5	132.113	1.16452	
30	2-Propanol	C67-63-0	C_3_H_8_O	60.1	540.6	112.085	1.21749	
31	Ethyl acetate	C141-78-6	C_4_H_8_O_2_	88.1	607.4	141.256	1.09931	monomer
32	Ethyl acetate	C141-78-6	C_4_H_8_O_2_	88.1	606.6	140.883	1.3438	dimer
33	2-Methylpropanol	C78-83-1	C_4_H_10_O	74.1	628.3	150.375	1.16871	monomer
34	2-Methylpropanol	C78-83-1	C_4_H_10_O	74.1	627.4	150.003	1.36194	dimer
35	(E)-2-Pentenal	C1576-87-0	C_5_H_8_O	84.1	748.2	224.229	1.11138	monomer
36	(E)-2-Pentenal	C1576-87-0	C_5_H_8_O	84.1	747.3	223.581	1.36555	dimer
37	1-Pentanol	C71-41-0	C_5_H_12_O	88.1	768.8	240.733	1.51103	
38	Furfural	C98-01-1	C_5_H_4_O_2_	96.1	849.4	324.875	1.08437	monomer
39	Furfural	C98-01-1	C_5_H_4_O_2_	96.1	847.9	323.18	1.33581	dimer
40	Gamma-butyrolactone	C96-48-0	C_4_H_6_O_2_	86.1	943.9	463.811	1.08519	monomer
41	Gamma-butyrolactone	C96-48-0	C_4_H_6_O_2_	86.1	941.4	459.34	1.30502	dimer
42	Methional	C3268-49-3	C_4_H_8_OS	104.2	913.2	409.263	1.0909	
43	Methyl hexanoate	C106-70-7	C_7_H_14_O_2_	130.2	920.8	422.676	1.28447	monomer
44	Methyl hexanoate	C106-70-7	C_7_H_14_O_2_	130.2	920.5	422.229	1.68557	dimer
45	Butyl acetate	C123-86-4	C_6_H_12_O_2_	116.2	816.5	288.54	1.61755	
46	6-Methyl-5-hepten-2-one	C110-93-0	C_8_H_14_O	126.2	990.8	547.119	1.16743	
47	2-Pentylfuran	C3777-69-3	C_9_H_14_O	138.2	993.6	552.086	1.25933	
48	Linalool	C78-70-6	C_10_H_18_O	154.3	1099.5	757.916	1.24181	

**Table 2:** Euclidean distances of different samples.

	[+] a-1	[+] a-2	[+] a-3	[+] b-1	[+] b-2	[+] b-3	[+] c-1	[+] c-2	[+] c-3
**[+] a-1**	0	244,780.261	349,867.703	10,298,521.170	10,797,214.659	10,768,595.551	11,414,096.203	10,833,142.541	10,653,051.491
**[+] a-2**	244,780.261	0	101,933.352	12,021,114.137	12,457,807.693	12,417,734.407	13,249,669.406	12,696,028.017	12,483,589.885
**[+] a-3**	349,867.703	101,933.352	0	12,578,385.513	13,066,327.608	13,032,175.961	13,504,086.200	12,940,298.655	12,735,107.148
**[+] b-1**	10,298,521.170	12,021,114.137	12,578,385.513	0	177,930.336	333,022.655	8,392,413.066	8,332,392.665	8,254,672.403
**[+] b-2**	1,079,7214.659	12,457,807.693	13,066,327.608	177,930.336	0	56,862.870	9,101,479.265	9,140,934.968	9,039,867.767
**[+] b-3**	10,768,595.551	12,417,734.407	13,032,175.961	333,022.655	56,862.870	0	8,995,502.234	9,098,087.522	8,991,662.351
**[+] c-1**	11,414,096.203	13,249,669.406	13,504,086.200	8,392,413.066	9,101,479.265	8,995,502.234	0	595,582.622	711,259.717
**[+] c-2**	10,833,142.541	12,696,028.017	12,940,298.655	8,332,392.665	9,140,934.968	9,098,087.522	595,582.622	0	15,974.500
**[+] c-3**	10,653,051.491	12,483,589.885	12,735,107.148	8,254,672.403	9,039,867.767	8,991,662.351	711,259.717	15,974.500	0

## Data Availability

Not applicable.

## Sample Availability

Samples of the compounds cistanches are available from the Inner Mongolia Sankou Biotechnology Co., Ltd.

## References

[1] Sun X., Li L., Pei J., Liu C., Huang L.F. (2020). Metabolome and transcriptome profiling reveals quality variation and underlying regulation of three ecotypes for Cistanche deserticola. Plant Mol. Biol..

[2] Li D.Q., Xu R., He X., Feng R., Xu C.Q., Liu T. (2021). Market Investigation and Study of Standard Grade of Cistanches Herba. Mod. Chin. Med..

[3] National Health Commission (2020). The two departments of the state issued a document: The pilot of 9 substances such as Ganoderma lucidum and tianma is both food and Chinese herbal medicine management. Edible Med. Mushrooms.

[4] Meng G., Yong H., Xin C., Zhi-Feng Z., Hong-Rui Z., Yan Z., He-Min L., Yu-Hai G. (2022). Distribution Characteristics of Mineral Elements in Different Types of Cistanche deserticola Y.C.Ma Were Anaylzed by ICP-MS. Spectrosc. Spectr. Anal..

[5] Jia W., Wen Y., Dong W., Xin-Hua Z., Lei G. (2021). Comparative studies of four Cistanche speices based on HPLC characteristic chromatogram. Chin. J. Pharm. Anal..

[6] Zhang J., Li C., Che Y., Wu J., Wang Z., Cai W., Li Y., Ma Z., Tu P. (2015). LTQ-Orbitrap-based strategy for traditional Chinese medicine targeted class discovery, identification and herbomics research: A case study on phenylethanoid glycosides in three different species of Herba Cistanches. RSC Adv..

[7] Liu W., Song Q., Cao Y., Xie N., Li Z., Jiang Y., Zheng J., Tu P., Song Y., Li J. (2019). From (1)H NMR-based non-targeted to LC-MS-based targeted metabolomics strategy for in-depth chemome comparisons among four Cistanche species. J. Pharm. Biomed. Anal..

[8] Xu C., Jia X., Xu R., Wang Y., Zhou Q., Sun S. (2013). Rapid discrimination of Herba Cistanches by multi-step infrared macro-fingerprinting combined with soft independent modeling of class analogy (SIMCA). Spectrochim. Acta A Mol. Biomol. Spectrosc..

[9] Xiong W.T., Gu L., Wang C., Sun H.X., Liu X. (2013). Anti-hyperglycemic and hypolipidemic effects of Cistanche tubulosa in type 2 diabetic db/db mice. J. Ethnopharmacol..

[10] Weirong B., Xufang Y., Lixing Z., Ruyi G., Yun W. (2020). Enzymatic Transformation of Verbascoside in Cistanche tubulosa. Food.

[11] Chen M., Cui G.-H., Xiao S.-P., Lin S.-F., Wu Z.-G., Huang Q. (2008). Preliminary study on variation pattern of Cistanche deserticola. China J. Chin. Mater. Med..

[12] Hong M., Rui C., Hong Y. (2006). The Comparative Anatomy Research on Vegetative Organs of Cistanche deserticola. J. Inn. Mong. Univ. Nat. Sci. Ed..

[13] Biao L. (2012). The Study on Effective Component Content in Cistanche. Master’s Thesis.

[14] Hou H., Liu C., Lu X., Fang D., Hu Q., Zhang Y., Zhao L. (2021). Characterization of flavor frame in shiitake mushrooms (Lentinula edodes) detected by HS-GC-IMS coupled with electronic tongue and sensory analysis: Influence of drying techniques. LWT.

[15] Yao W., Cai Y., Liu D., Zhao Z., Zhang Z., Ma S., Zhang M., Zhang H. (2020). Comparative analysis of characteristic volatile compounds in Chinese traditional smoked chicken (specialty poultry products) from different regions by headspace-gas chromatography-ion mobility spectrometry. Poult. Sci..

[16] Wu Y., Liang S., Zheng Y., Zhang M. (2020). Volatile Compounds of Different Fresh Wet Noodle Cultivars Evaluated by Headspace Solid-Phase Microextraction-Gas Chromatography-Mass Spectrometry. An. Acad. Bras. Cienc..

[17] Wei X.-F., Ma X.-L., Cao J.-H., Sun X.-Y., Fang Y.-L. (2018). Aroma characteristics and volatile compounds of distilled Crystal grape spirits of different alcohol concentrations: Wine sprits in the Shangri-La region of China. Food Sci. Technol..

[18] Han Y., Wang C., Zhang X., Li X., Gao Y. (2022). Characteristic volatiles analysis of Dongbei Suancai across different fermentation stages based on HS-GC-IMS with PCA. J. Food Sci..

[19] Fan X., Jiao X., Liu J., Jia M., Blanchard C., Zhou Z. (2021). Characterizing the volatile compounds of different sorghum cultivars by both GC-MS and HS-GC-IMS. Food Res. Int..

[20] Yang L., Liu J., Wang X., Wang R., Ren F., Zhang Q., Shan Y., Ding S. (2019). Characterization of Volatile Component Changes in Jujube Fruits during Cold Storage by Using Headspace-Gas Chromatography-Ion Mobility Spectrometry. Molecules.

[21] Li M.Q., Yang R.W., Zhang H., Wang S.L., Chen D., Lin S.Y. (2019). Development of a flavor fingerprint by HS-GC-IMS with PCA for volatile compounds of Tricholoma matsutake Singer. Food Chem..

[22] Jampaphaeng K., Ferrocino I., Giordano M., Rantsiou K., Maneerat S., Cocolin L. (2018). Microbiota dynamics and volatilome profile during stink bean fermentation (Sataw-Dong) with Lactobacillus plantarum KJ03 as a starter culture. Food Microbiol..

[23] Yang F., Liu Y., Wang B., Song H., Zou T. (2021). Screening of the volatile compounds in fresh and thermally treated watermelon juice via headspace-gas chromatography-ion mobility spectrometry and comprehensive two-dimensional gas chromatography-olfactory-mass spectrometry analysis. LWT.

[24] Dong W., Tan L., Zhao J., Hu R., Lu M. (2015). Characterization of Fatty Acid, Amino Acid and Volatile Compound Compositions and Bioactive Components of Seven Coffee (Coffea robusta) Cultivars Grown in Hainan Province, China. Molecules.

[25] Hu X., Wang R., Guo J., Ge K., Li G., Fu F., Ding S., Shan Y. (2019). Changes in the Volatile Components of Candied Kumquats in Different Processing Methodologies with Headspace-Gas Chromatography-Ion Mobility Spectrometry. Molecules.

[26] Feng D., Wang J., He Y., Ji X.J., Tang H., Dong Y.M., Yan W.J. (2021). HS-GC-IMS detection of volatile organic compounds in Acacia honey powders under vacuum belt drying at different temperatures. Food Sci. Nutr..

[27] Wu Z., Chen L., Wu L., Xue X., Zhao J., Li Y., Ye Z., Lin G. (2015). Classification of Chinese Honeys According to Their Floral Origins Using Elemental and Stable Isotopic Compositions. J. Agric. Food Chem..

[28] Zhou S.-Q., Feng D., Zhou Y.-X., Zhao J., Zhao J.-Y., Guo Y., Yan W.-J. (2022). HS-GC-IMS detection of volatile organic compounds in cistanche powders under different treatment methods. LWT.

